# High resolution mapping and development of diagnostic KASP markers for FHB resistance QTL on chromosome 1B in an elite soft red winter wheat cultivar ‘Jamestown’

**DOI:** 10.1007/s11032-026-01680-7

**Published:** 2026-05-28

**Authors:** Lovepreet Singh, Anmol Kajla, Prem Kumar Ganesan, Yanhong Dong, Gina Brown Guedira, Jared Smith, Vijay Tiwari, Nidhi Rawat

**Affiliations:** 1 Department of Plant Science and Landscape Architecture, University of Maryland College Park, College Park, MD 20742 USA; 2 Department of Plant Pathology, University of Minnesota, St. Paul, Minnesota 55108 USA; 3 USDA-ARS, North Carolina State University, Raleigh, NC 27695 USA

**Keywords:** Fusarium Head Blight, DON, High resolution mapping, KASP assays, Jamestown, Breeding

## Abstract

Fusarium Head Blight (FHB) is a serious disease of wheat, causing yield and quality losses to the grain. Genetic resistance is one of the most important strategies to manage FHB. QTL from unadapted genetic backgrounds, such as wild relative species and exotic landraces, have been used in wheat breeding lines across the world. However, deployment of such sources poses problems associated with linkage drag, that impede their quick deployment in cultivars. Previously, we reported mapping of an FHB resistance QTL on chromosome 1B of an elite soft red winter wheat (SRW) cultivar Jamestown using Recombinant Inbred Lines (RIL) populations. In this work, we developed a high-resolution mapping population with 475 individuals by crossing resistant and susceptible RILs. Genome-specific KASP markers were developed and used to genotype the population. Phenotyping, followed by progeny testing of the recombinants, reduced the genetic interval to a 3 Mb region predicted to contain 17 high-confidence genes in the corresponding region in the Chinese Spring reference genome ver 1.0. The KASP marker flanking the 1B QTL region was validated on the SRW breeding germplasm of the Eastern US, which showed improved correlation over the previous markers used for MAS of this QTL. This work will facilitate breeding of 1B_FHB QTL from Jamestown to wheat varieties widely.

**Supplementary Information:**

The online version contains supplementary material available at 10.1007/s11032-026-01680-7.

## Introduction

Wheat (*Triticum aestivum* L.) is the staple food crop of about 35% of the human population globally and contributes to one fifth of the calories and protein consumed by human population (Alomari et al. 2023; FAOSTAT, 2026). Hence, sustainable wheat production is critical for global food security. Fusarium Head Blight (FHB), caused by *Fusarium graminearum*, is one of the most devastating diseases of wheat worldwide, leading to significant yield losses (Savary et al. 2019). FHB causes billions of dollars of crop losses every year (Friskop et al. 2025; McMullen et al. 2012). In addition to posing a threat to food security, FHB-associated mycotoxin (primarily deoxynivalenol, DON) contamination inflicts a serious risk to human and livestock health. Crop management practices and fungicide applications are partially effective in controlling FHB in wheat. The cultivation of FHB-resistant varieties is the most effective, economically sustainable and environmentally friendly strategy to manage FHB in wheat (McMullen et al. 2012). Therefore, breeding for FHB resistance remains one of the major goals of wheat breeding programs across the globe (Li et al. 2022).

FHB resistance is controlled by multiple loci and exhibits high Genotype × Environment interaction (G× E) (Mesterházy 1995). In wheat, quantitative trait loci (QTLs) for FHB resistance have been reported on almost all chromosomes (Buerstmayr et al. 2009, 2020). However, most of them are minor-effect QTLs with low stability. Only a few major effect QTLs have been identified in wheat. To date nine QTLs have been Mendelized and assigned with gene names including: *Fhb1* on 3BS derived from *T. aestivum* variety Sumai 3 (Cuthbert et al. 2006), *Fhb2* on 6BS also from Sumai3 (Cuthbert et al. 2007), *Fhb3* on 7 A derived from *Leymus racemosus* (Qi et al. 2008), *Fhb4* on 4B derived from *T. aestivum* cv. Wangshuibai (Xue et al. 2010), *Fhb5* on 5 A derived from Wangshuibai (Xue et al. 2011), *Fhb6* on 1 A derived from *Elymus tsukushiensis* (Cainong et al. 2015), *Fhb7* on 7D derived from *Thinopyrum ponticum* (Guo et al. 2015), *Fhb8* also on 7D derived from Wangshuibai (Wang et al. 2024), and *Fhb9* on 2DL derived from Shi4185 (Zhang et al. 2025). Since most of these major effect resistance sources are derived from Asian landraces or wild relatives, their introgression into contemporary locally adapted cultivars for the U.S. is often challenging due to associated linkage drag or background interactions (Buerstmayr et al. 2020; Liu et al. 2013). Thus, it is highly desirable to characterize and utilize native sources of genetic resistance derived from adapted germplasm (Liu et al. 2013; Carpenter et al. 2020) that can be readily deployed in cultivars.

Jamestown (PI 653671, abbreviated as JT henceforth) is a widely adapted, high-yielding, moderately FHB-resistant soft red winter wheat (SRWW) cultivar developed by a cross between the ‘Roane’ (PI 612958) and Pioneer Brand 2691 (PI 590941) by the Virginia Tech wheat breeding program in the USA (Griffey et al. 2010). JT has been used in various Southern and Mid-Atlantic wheat breeding programs of the US as a donor for FHB resistance (Griffey et al. 2010). A study by Ghimire et al. (2024) showed the resistance of the cultivar JT against four isolates of *F. graminearum* with least severity caused by NIV (nivalenol) producing isolates. Two FHB resistance QTLs, *QFHB.vt-1B.1* and *QFHB.vt-1B.2*, were identified and characterized on the long arm of chromosome 1B of JT and the QTLs were validated in three different mapping populations (Carpenter et al. 2020).

Marker-assisted selection (MAS) is highly desirable for FHB resistance, given the challenges associated with phenotyping, high G×E interactions and the requirement of adult plant screening (Buerstmayr et al. 2009). Increasingly available genomics resources are providing new tools for marker development that were not available before in wheat (Consortium (IWGSC) et al. 2018; Ramírez-González et al. 2018; He et al. 2019; Walkowiak et al. 2020). Although Kompetitive allele-specific polymerase chain reaction (KASP) assays for MAS were developed for Jamestown FHB QTLs, they were mapped across a significantly large genetic interval (Carpenter et al. 2020). Therefore, this work was subsequently conducted to refine the JT FHB 1B QTL mapping interval region, which will be useful for its precise MAS in breeding programs and eventually cloning the underlying gene.

The objectives of this study were to perform high-resolution mapping of the FHB resistance QTL on chromosome 1B of SRWW cultivar Jamestown and develop diagnostic KASP assays for precise MAS for pyramiding of FHB resistance genes and alleles into elite wheat germplasm.

## Materials and methods

### Plant materials

A previously developed recombinant inbred line (RIL) population at the F_8_ generation, developed from parents Pioneer Brand 25R47 (PR- susceptible) and Jamestown (JT- moderately resistant) cultivars, was used to generate genetic material for this study (Carpenter et al. 2020). A high-resolution mapping population was developed by crossing one highly susceptible RIL (PJT_62) and one highly resistant RIL (PJT_72), which showed contrasting genotypes for the *QFHB.vt-1B.1* locus, followed by the selfing of the F_1_ plants. Four hundred seventy-five F_2_ individuals were genotyped and phenotyped for FHB and only two plants were found to have a recombination breakpoint between the flanking markers. To reconfirm the phenotypes, F_2:3_ and F_3:4_ families of the selected F_2_ and F_3_ plants, respectively, were used for genotypic and phenotypic analyses of the *QFHB.vt-1B.1* locus. Plants were grown in greenhouse conditions with a day temperature of 25 ± 2 °C and night temperature at 22 ± 2 °C. Timely irrigation, fertilizer and insecticide treatments were applied.

### Fungal inoculum preparation and inoculations

A highly aggressive strain of *Fusarium graminearum*, GZ3639, was used for all the greenhouse experiments in the study. The inoculum was prepared in Mung Bean Broth (MBB). MBB was prepared by steeping 20 g of mung bean seeds in 500 ml of hot water for 40 min. The collected filtrate was autoclaved and cooled. For macroconidia production, two mycelial plugs from a one-week-old PDA culture were added per 50 ml of MBB and incubated at 28 °C and 250 rpm for 7–10 days. The spore culture was filtered through cheese cloth and macroconidia concentration was measured using hemocytometer. The macroconidia concentration was adjusted to 1 × 10^5^ spores/ ml by diluting with sterile water. The spores were harvested freshly before each inoculation.

Inoculations were performed at pre-anthesis stage to anthesis stage. Tenth and eleventh spikelets counting from the base of the spikes were marked with a black permanent marker, and 10 µl of inoculum was injected between the lemma and palea of the florets (one floret/spikelet) using a pipette. Clear plastic bags sprayed with two quirts of water were used to cover the inoculated spikes for 72 h to provide high humidity for optimal fungal growth. The temperature was kept the same as that during the growth of the plants (25 ± 2 °C at day and 22 ± 2 °C at night).

### Disease assessment

Two disease assessment parameters: FHB severity and DON content, were measured. FHB severity was measured as the Percentage of symptomatic spikelets (PSS). PSS was measured by recording the number of infected spikelets below the inoculation point, including the tenth spikelet. Measurements were taken at 28 days post-inoculation (dpi).

Inoculated spikes from each plant were harvested at maturity and manually threshed. Seeds from all inoculated spikes of each genotype per plant were pooled and ground to a fine powder. The ground powder was redistributed to create three technical replicates and used for measuring DON content. The DON content measurements were performed at the US Wheat and Barley Scab Initiative’s DON-testing laboratory at the University of Minnesota using gas chromatography-mass spectrometry (GC-MS) as previously described (Singh et al. 2021).

### Marker development and genotyping

For marker development, the previously reported flanking markers of the combined *QFHB.vt-1B.1*, and *QFHB.vt-1B.2*. (Carpenter et al. 2020), IWB43992 and IWB7443, were localized on the reference genome RefSeq v 1.1 (International Wheat Genome Sequence Consortium 2018) at the long arm of 1B chromosome (Consortium (IWGSC) et al. 2018). The combined region spanned from 330,214,153 bp to 476,880,056 bp with total length of approximately 147 Mb. Genome specific PCR primers for chromosome 1B were designed using GSP software with default settings (Wang et al. 2016) or manual alignments with SnapGene software and screened for polymorphism. Genome specificity was confirmed using the DNA of the Chinese Spring nulli-tetrasomic line for the 1B chromosome (N1B-T1A) as a control. A total of thirteen 1B-specific markers with one or more Single Nucleotide Polymorphism (SNP) between JT and P were identified and used for fine-mapping (Supplementary Table 1).

Leaf tissue was collected at the three-leaf stage into 1.2 ml 96-deep well plates. After snap-freezing in liquid nitrogen, the tissue was ground into a fine powder using metal beads with TissueLyser II (Qiagen, Germantown, MD). DNA was extracted using BioSprint 96 DNA Plant Kit (Qiagen, Germantown, MD) and KingFisher Flex system (Thermo Scientific, Waltham, MA) following the manufacturer’s protocol. The polymerase chain reactions (PCR) were performed in 25 µl reaction volume including 5 µl of 5X MyTaq buffer (Meridian Bioscience, Cincinnati, OH), 2 µl of each forward and reverse primers (4 µM/ µl), 0.1 µl of My Taq DNA Polymerase (Meridian Bioscience, Cincinnati, OH) and 50 ng of DNA template. A touch down PCR profile from 64 °C to 58 °C (95 °C for 5 min, 7 cycles of 95 °C for 45 s, 64 –58 °C for 45 s with a decrease of 1 °C per cycle, 72 °C for 1 min, followed by 27 cycles of 95 °C for 45 s, 58 °C for 45 s, 72 °C for 1 min, and a final extension of 72 °C for 7 min) was run on T100 thermal cycler (Bio-rad Laboratories Inc., CA). PCR products were resolved with agarose gel electrophoresis to check amplification and specificity. PCR products were cleaned up with ExoSAP-IT reagent and then sequenced in-house using BigDye Terminator v3.1 cycle sequencing Kit and 3730xl DNA Analyzer (Thermo Scientific, Waltham, MA).

### KASP marker assay development

Three 1B genome-specific SNP markers in the target interval were used to develop KASP assays. The guidelines by Makhoul et al. ( 2020) were followed to design primers maintaining both genome and allele specificity. The allele-specific SNP and genome-specific SNP were kept at the 3’ end of the reverse and common forward primers, respectively. The FAM and HEX tags were appended to the 5’ end of the allele-specific reverse primers. Ten microliters of KASP assay reaction included 5 µl of LGC KASP master mix, 0.14 µl of primer assay mix, and 5 µl of DNA template (10 ng/ µl). The assay mix included 12 µM of each forward allele-specific primer and 30 µM of common forward primer. The reactions were run on the BIO-RAD CFX96 Real-Time System-C1000 Touch cycler, using the all-channels option. The running profile was as follows: 94 °C for 15 min, 10 cycles of 94 °C for 20 s, 65 –57 °C for 60 s with a decrease of 0.8 °C per cycle, followed by 39 cycles of 94 °C for 20 s and 57 °C for 60 s, with a subsequent plate reading step at 35 °C for 60 s. The allele calling was performed using the allele discrimination mode of the BIO-RAD CFX Manager 3.1 software.

The KASP markers developed and used in mapping were validated on one hundred fifty-five public breeding program entries and checks in the 2025 Uniform Eastern and Southern Soft Red Winter Wheat Nurseries (UESRWWN and USSRWWN) and 2025 Northern, Preliminary Northern, and Southern Uniform Winter Wheat Scab Nurseries (NUWWSN, PNUWWSN, and SUWWSN).

### Statistical analyses and genetic mapping

Data were analyzed using GraphPad Prism version 9.3.1 and R statistical analysis software. In most cases, PSS and DON data were Log transformed to stabilize variances. One-way analysis of variance (ANOVA) was performed, followed by Fisher’s LSD to detect statistically significant differences between genotypes. Linkage map in F_2_ population was prepared using IciMapping 4.0 software (http://www.isbreeding.net). Grouping was performed at LOD of 3.0 and genetic distance between the markers was calculated using Kosambi mapping function. Maps were drawn using the software MapChart 2.2 (http://www.earthatlas.mapchart.com). Single marker regression analyses in the F_2_ population were performed using the R package ggpubr.

## Results

### Refining the *QFHB.vt-1B* region by identification and phenotyping of critical RILs

**Fig. 1:**
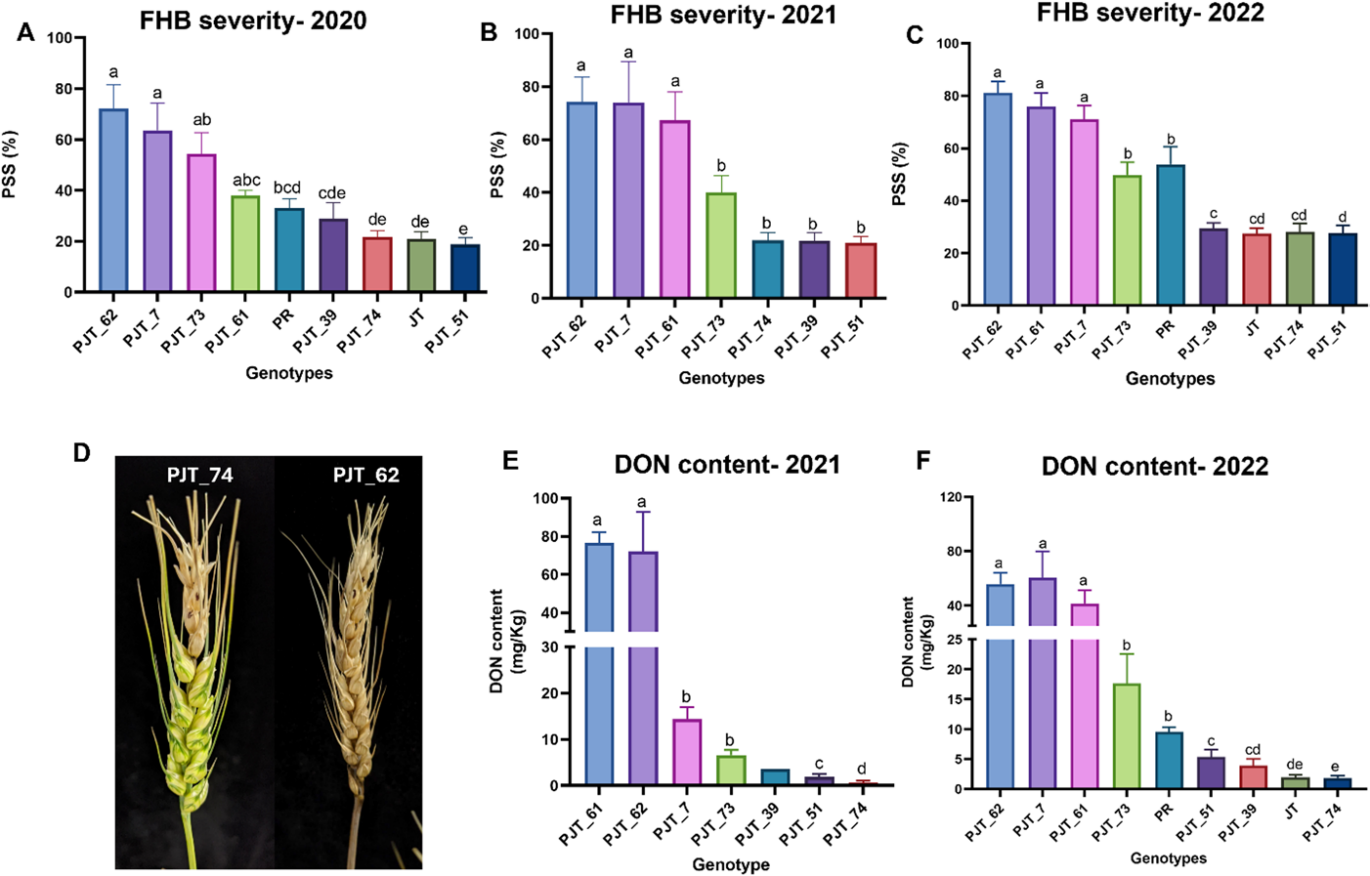
Phenotyping of critical RILs. Mean PSS ± SE from 2020 (**A**), 2021 (**B**), and 2022 (**C**). FHB symptoms in two contrasting RILs: PJT_74 and PJT_62 which were used as parents of the high-resolution mapping population (**D**). Mean DON content ± SE during spring 2021 (**E**) and 2022 (**F**). Bars with same letter in each panel are not statistically significantly different from each other at p value < 0.05

The combined *QFHB.vt-1B* region was previously mapped to an 18.8 cM region on the peri-centromeric region of the long arm of chromosome 1B flanked by marker IWB43992 (74.4 cM) and IWB7443 (55.6 cM) in Pioneer25R47/ JT population (Carpenter et al. 2020). Physical location of these flanking markers on the long arm of 1B chromosome revealed a 147 Mb region from 330 to 477 Mb coordinates to contain the QTL. We developed a total of thirteen 1B genome-specific sequence-based SNP markers spanning a 160 Mb region downstream of IWB7443 (Supplementary Table 1). After genotyping the RIL population, we identified five RILs with break points in the 147 Mb region: PJT_39, PJT_73, PJT_61, PJT_62, and PJT_7. This set of critical RILs, in addition to resistant PJT_74 and PJT51 (having JT alleles throughout were used as controls), was robustly phenotyped for FHB response in greenhouse conditions for three seasons (in 2020, 2021 and, 2022). In addition to assessing disease severity by measuring PSS, DON content was measured for two seasons. In all three seasons, genotypes varied significantly (p value < 0.001) for PSS (Fig. 1). Mean PSS ranged from 18 to 72% in 2020 (Fig. 1A), from 21 to 74% in 2021 (Fig. 1B), and 27 to 81% in 2022 (Fig. 1C). Statistical analysis for the mean differences enabled classification of the critical RILs into two phenotypic classes. PJT_62, PJT_61, PJT_7, and PJT_73 were identified as susceptible RILs with significantly (p value < 0.05) higher PSS than the resistant parent JT. On the other hand, PJT_39 as well as two other RILs (PJT_51 and PJT_74) with JT alleles for all the tested markers had significantly (p value < 0.05) lower PSS value than the susceptible RILs and susceptible parent PR, and were identified as resistant RILs. PJT 62, having high FHB susceptibility was used in all experiments as the susceptible control (Fig. 1D).

Similar, trends were observed for the DON content. Genotypes varied significantly (p value < 0.001) for DON content in Spring 2021 and 2022. In 2021, mean DON content varied from 0.7 to 77 mg/Kg (Fig. 1E). In 2022, mean DON content ranged from 1.8 to 60 mg/kg (Fig. 1F). In agreement with the PSS, the four susceptible RILs (PJT_62, PJT_61, PJT_7, and PJT_73) had significantly higher DON content than the three resistant RILs (PJT_39, PJT_51, and PJT_74).

The PSS and DON content of some of the susceptible and resistant RILs was more contrasting than those of the parents (PR and JT) of the RIL population (Fig. 1), suggesting them to be transgressive segregants.

**Fig. 2:**
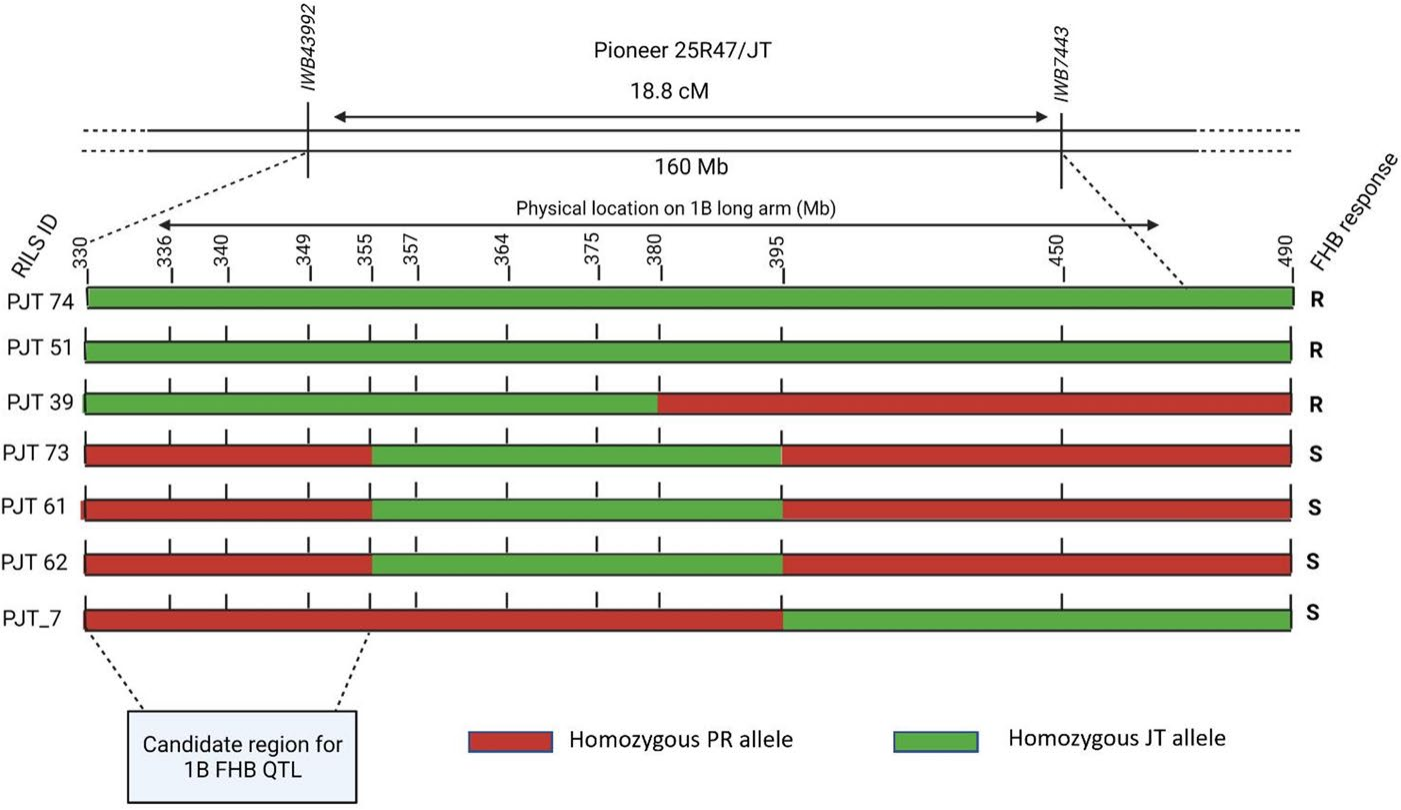
Mapping of 1B FHB QTL using critical RILs. The presence of JT alleles in susceptible RIL PJT_7 in the region beyond 395 Mb, and presence of PR alleles in resistant RIL PJT_39 in the region beyond 380 Mb delimited the resistance locus to the region before 380 Mb. Absence of JT alleles in the interval from 330 Mb to 355 MB in susceptible RILs PJT_61, PJT_62, and PJT_73 narrowed down the target region between 330 Mb as the left boundary and 355 Mb as the right boundary, reducing the mapping interval to 25 Mb. Genotyping scores were assigned based on the sequencing based GSP SNP markers. R: resistant to FHB and having low DON; S: susceptible to FHB and having high DON

Integrating the phenotypic and genotypic analyses of the RILs having recombination in the QTL region the 147 Mb target region was resolved further (Fig. 2). The presence of JT alleles in susceptible RIL PJT_7 in the region beyond 395 Mb, and presence of PR alleles in resistant RIL PJT_39 in the region beyond 380 Mb delimited the resistance locus to the region before 380 Mb. Susceptible RIL PJT_61, PJT_62, and PJT_73 each possessed JT alleles in the interval from 355 Mb to 395 MB. In contrast, JT alleles were present in the interval from 330 Mb to 355 Mb in the resistant RIL PJT39, PJT 51 and PJT74. These data narrowed down the target region between 330 Mb as the left boundary and 355 Mb as the right boundary, reducing the mapping interval to 25 Mb (Fig. 2).

### Phenotypic and genotypic analyses of the F_2_ mapping population

RILs PJT_62 and PJT_74 consistently exhibited highly contrasting responses to FHB (Fig. 1F) and were therefore used to develop a high-resolution mapping population. Four-hundred seventy-five F_2_ individuals of the PJT_62/PJT_74 population were phenotyped for PSS. The mean PSS of the F_2_ population was 43.3% with a range of 18 to 100%. The frequency distribution of mean PSS of F_2_ individuals was continuous, indicating that PSS is controlled by multiple QTLs in this population (Supplementary Fig. 1A).

Three KASP marker assays KASP330, KASP340, and KASP355 were developed to genotype the F_2_ population (Table 1). All three KASP assays clearly differentiated homozygous resistant, homozygous susceptible, and heterozygous genotypes (Supplementary Fig. 2). The F_2_ genotyping data was used to construct a genetic map of the three KASP markers. The marker order in the genetic map was in agreement with the physical location of markers (Supplementary Fig. 1B). Subsequently, F_2_ genotyping and phenotyping data was used to perform single marker regression analysis. All three markers 330 (*r* = 0.11, *p value* = 0.016), 340 (*r* = 0.15, *p value* = 0.0011), and 355 (*r* = 0.15, *p value* = 0.0011) showed weak but positive and significant association with the PSS phenotype.

**Table 1:** Sequences of KASP primers developed in this study

Primer	Sequence 5'-3'a
KASP330-R	**GAAGGTGACCAAGTTCATGCT**TGCTTAATGCTGACATGGGTAAAAAA
KASP330-S	**GAAGGTCGGAGTCAACGGATT**TGCTTAATGCTGACATGGGTAAAAAG
KASP330-common	GTCACTTAACAGGAGAGATAGAAGAACAA
KASP340-R	**GAAGGTGACCAAGTTCATGCT**AGTGTTCCCTATTTTGAGCAAAAGC
KASP340-S	**GAAGGTCGGAGTCAACGGATT**AGTGTTCCCTATTTTGAGCAAAAGA
KASP340-common	ACATTATGAATTTTCACGTAAACAACGC
KASP355-R	**GAAGGTGACCAAGTTCATGCT**ATCAACGTGCAACTAATACATCTTACT
KASP355-S	**GAAGGTCGGAGTCAACGGATT**ATCAACGTGCAACTAATACATCTTACC
KASP355-common	TCACTAGTAATCGTGTATGTGCA

^a^Allele and genome specific SNPs are underlined and italicized

FAM and HEX sequences are highlighted in bold font

### Mapping of 1B FHB QTL to a 3 Mb physical region using KASP markers

**Fig. 3:**
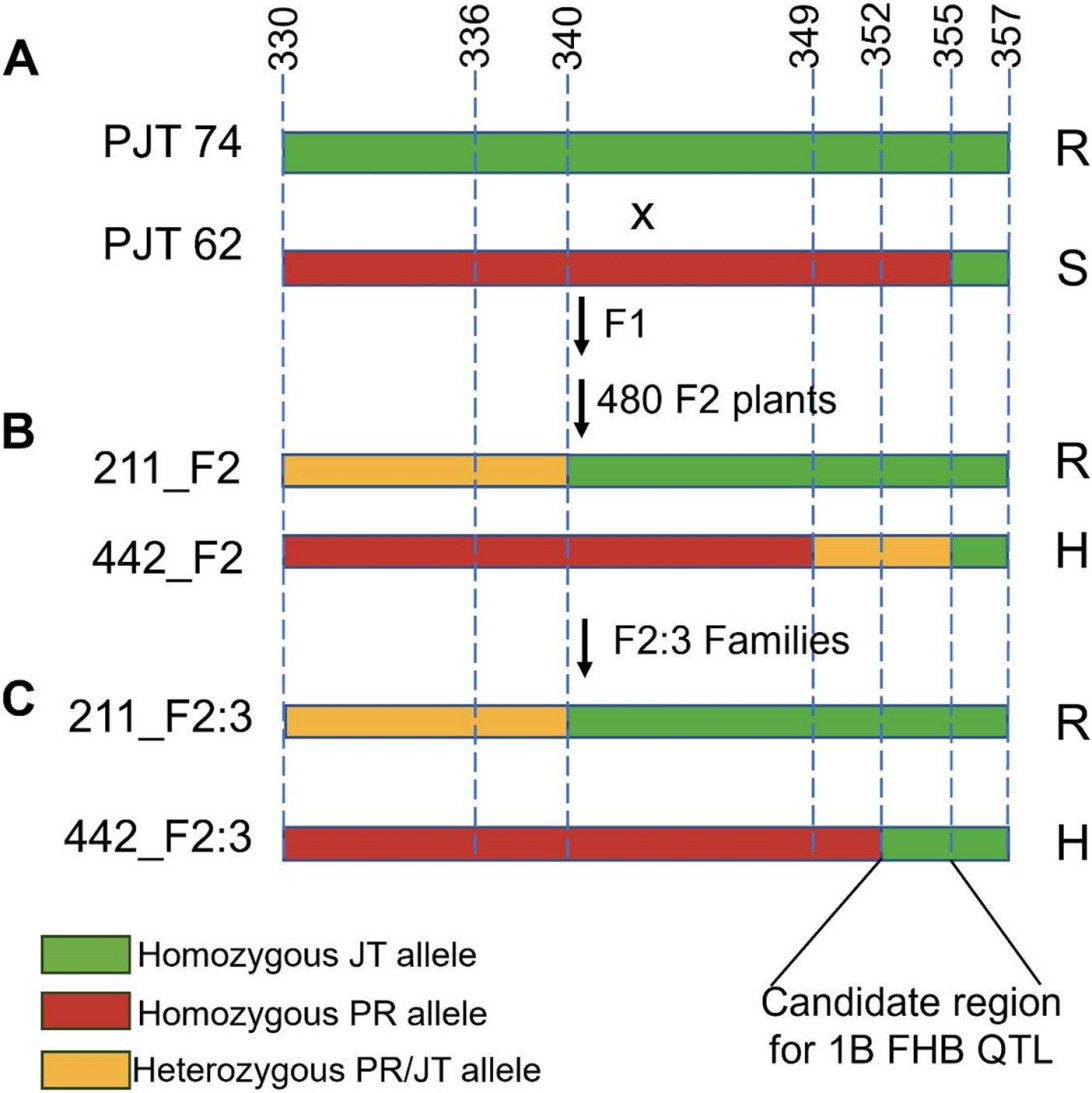
Mapping of 1B FHB QTL to a 3 Mb interval. **A**)- High resolution mapping population was developed by crossing highly susceptible RIL PJT_62 and highly resistant RIL PJT_74. **B**)-F_2_ population and **C**)- F_2:3_ progenies were genotyped using KASP assays in the 2 Mb target region and genotypes were confirmed using sequencing based GSP SNP markers. Phenotypic response of plants to FHB on the right side of all the lines- R: resistant to FHB and having low DON; S: susceptible to FHB and having high DON

To further narrow down the 25 Mb QTL region, recombinants were screened for it in the PJT_62/PJT_74 high resolution population. Out of the 475 F_2_ individuals, only two plants had recombination in the target region. The plant 211_F_2_ was heterozygous for KASP330 and KASP340 markers and homozygous resistant for KASP355 marker (Fig. 3B). The 442_F_2_ had a homozygous susceptible genotype for KASP330 and KASP340 markers, whereas it was heterozygous for the KASP355 marker. More genome-specific sequence-based markers were developed at 349, 352, and 357 Mb for further fine-mapping the recombinants. Genotyping with these markers identified the breakpoint for 211_F_2_ at 340 Mb region and for the 442_F_2_ individual at 352 Mb region, while 357 Mb marked the right boundary for both the recombinants (Fig. 3B).

**Fig. 4:**
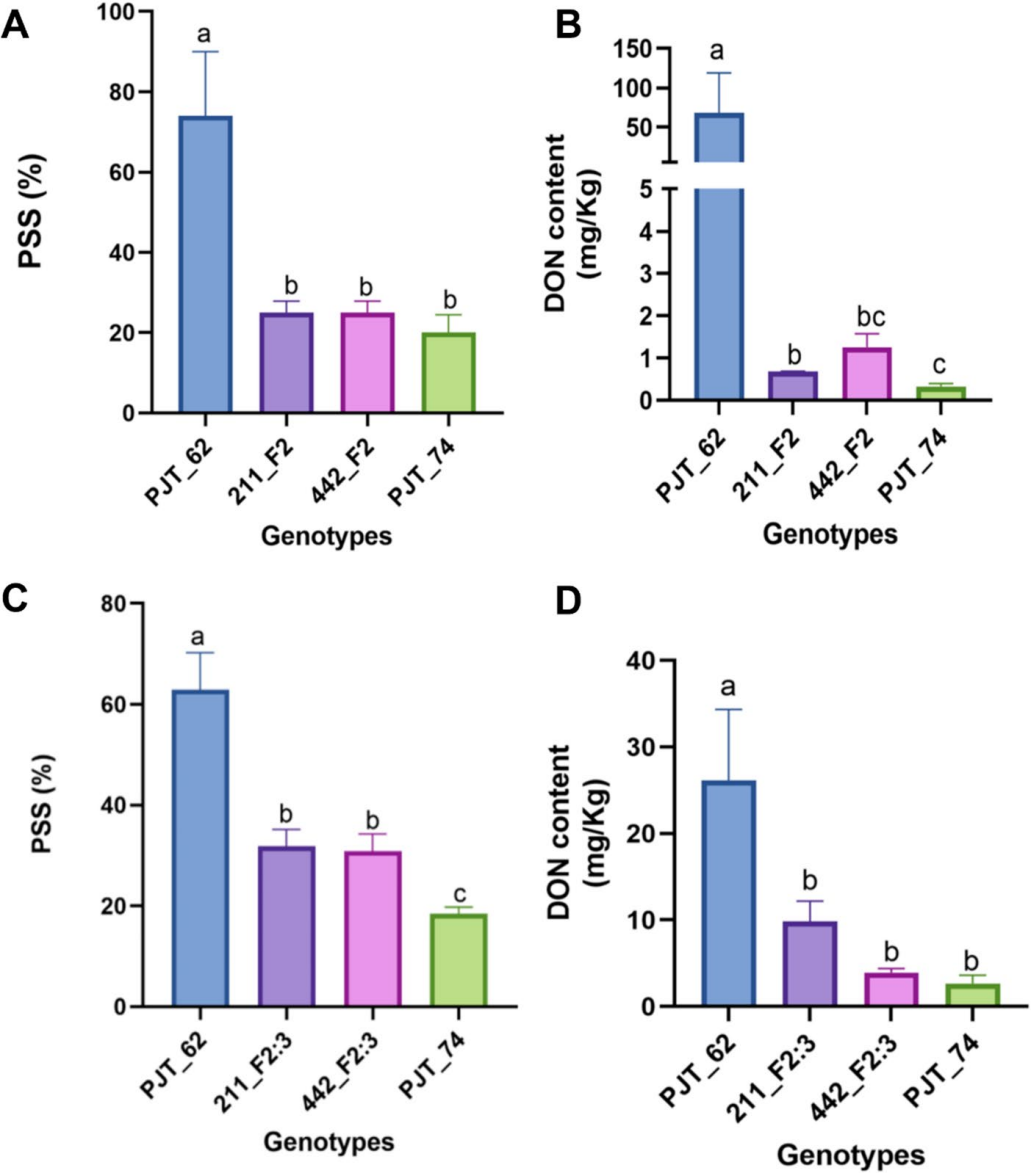
Phenotyping of recombinant F_2_ individuals and their homozygous recombinant F_2:3_ progenies. (**A**) Mean PSS ± SE of the selected F_2_ plants and the parents (PJT_62 and PJT_74). (**B**) DON content ± SE of F_2_ plants and the parents (PJT_62 and PJT_74). (**C**) Mean PSS ± SE of homozygous recombinant F_2:3_ progenies and the parents. (**D**) DON content ± SE of homozygous recombinant F_2:3_ progenies and the parents. Data were log-transformed before the statistical analysis. Bars with same letter in each panel are not statistically significantly different from each other at p value < 0.05

Subsequently, F_2:3_ families of 211_F_2_ and 442_F_2_ individuals were screened for homozygous recombinants. Eight and eleven homozygous recombinants were identified in the target 25 Mb region for F_2:3_ families of 211_F_2_ and 442_F_2_ plants, respectively. The selected F_2_ individuals and F2:3 families were phenotyped for FHB response and DON content and genotyped for markers at 349_1B_FHB, 352_1B_FHB, and 355_1B_FHB (Fig. 4). Genotypic markers showed resistant alleles of 442_F_2:3_ to be homozygous resistant at 355_1B_FHB marker (Fig. 3C). The recombinant F_2_ plants and their F_2:3_ families had statistically significantly (*p* < 0.05) lower mean PSS and mean DON content compared to the susceptible parent PJT_62 (Fig. 4). These results were consistent with the DON content analysis of F_3:4_ progenies of the selected F_2_ recombinants (Supplementary Fig. 4). Therefore, genotypic, and phenotypic analysis of recombinant F_2_ individuals narrowed the target region to 3 Mb between 352 and 355 Mb region (Fig. 3). Within this 3 Mb target region, there are a total of 17 putative high-confidence genes based on the IWGSC ref v 1.1 database.

### Validation of the KASP markers on the US soft red winter wheat breeding germplasm

KASP markers for 352, 355, and 357 Mb markers were evaluated on entries originating from public breeding programs by the USDA-ARS Eastern Regional Small Grains Genotyping Labin the 2025 US Eastern Soft Red Winter Nursery, US Southern Soft Red Winter Nursery, Uniform Southern Soft Red Winter Wheat Nursery, Northern Uniform Winter Wheat Scab Nursery, Preliminary Northern Uniform Winter Wheat Scab Nursery, and Southern Uniform Winter Wheat Scab Nursery. Markers were able to reliably distinguish between homozygous and heterozygous/heterogeneous clusters on sample DNA isolated from five-seed tissue bulks (Supplementary Fig. 4, Supplementary Table 2). Presence or absence of Jamestown FHB 1B resistance was assigned based on haplotypes of alleles across the three markers, with presence of one or more susceptible alleles resulting in assignment of a non -resistant haplotype. The presence of one or more heterozygous calls across an otherwise resistant haplotype resulted in assignment of a “het” haplotype (i.e., heterozygous or heterogeneous). Marker data revealed that the presence of Jamestown FHB 1B is common in the tested germplasm, with 44.5% of lines exhibiting the homozygous resistant haplotype, and 47.7% of lines showing allele combinations denoting susceptible haplotypes.

## Discussion

Deploying genetic resistance is the most economical and sustainable strategy to control FHB in wheat. Given the genetics of FHB resistance with high GxE interaction, phenotypic evaluation of FHB resistance needs to be repeated over multiple environments. Therefore, phenotypic selection to improve FHB resistance is very time-consuming and resource-intensive (Bai and Shaner 2004). Employing MAS using diagnostic markers can significantly improve selection efficiency for improving FHB resistance. For developing diagnostic markers, high-resolution mapping of the target region is a prerequisite. Previously, two novel QTLs were identified on chromosome 1B of the high yielding and moderately FHB resistant cultivar “Jamestown” (Carpenter et al. 2020). In this study, we developed high resolution mapping populations in the QTL *QFHB.vt-1B.1* region, and by integrating the genotypic and phenotypic data of recombinants in the target interval, we placed the QTL in a 3 Mb region between 352 and 355 Mb on chromosome 1B of Jamestown. Diagnostic KASP markers were developed for use in breeding programs.

Genotyping of the JT/ PR RIL population with genome specific polymorphic markers in the 147 Mb physical region of the QTL resulted in the identification of a critical set of RILs with recombination events in the region. Comparative analysis of genotype and robust FHB and DON phenotype data narrowed down the target region to 25 Mb, between 330 and 355 Mb. The target 25 Mb region is located very close to the centromere, and low recombination rates are reported in the pericentromeric region of wheat (Saintenac et al. 2009; Jordan et al. 2018). To overcome this barrier, we developed a high-resolution mapping population between two RILs contrasting in their genotype and phenotype in this 25 Mb region. Using the genotype and phenotype data of recombinants, Jamestown FHB QTL interval was further refined to 3 Mb region between 352 and 355 Mb.

KASP marker assays are a breeder-friendly, cost-effective marker platform for high-throughput SNP genotyping of breeding materials (Semagn et al. 2014). KASP marker assays are being used for successful pyramiding of various QTLs for diverse traits in wheat breeding (Kaur et al. 2020; Jan et al. 2025; Wang et al. 2025; Tian et al. 2025). Development of co-dominant KASP markers is challenging in polyploids like wheat compared to diploid crops since genome specificity needs to be taken in consideration while developing KASP assays (Allen et al. 2013; Makhoul et al. 2020). In this study, we developed three 1B genome-specific KASP marker assays. All three KASP assays clearly differentiated between homozygous and heterozygous individuals and followed expected genotyping ratio (1:2:1) in the F_2_ population. These KASP assays will enable precise MAS for Jamestown QTL *QFHB.vt-1B.1*. The 3 Mb target region has 17 high-confidence genes. Further analysis and fine mapping will be required to identify the underlying candidate gene for FHB resistance.

In conclusion, we refined the native resistance to FHB severity and DON in the soft red winter wheat cultivar Jamestown to a 3 Mb region. The breeder-friendly diagnostic KASP markers developed in the study would be a very useful resource for pyramiding QTLs for enhancing FHB resistance widely. Hence, the resources developed in this study would enable efficient deployment of Jamestown FHB QTL and facilitate the efforts to identify underlying causal genes.

## Electronic Supplementary Material

Below is the link to the electronic supplementary material.

Supplementary Material 1 (XLXS 16.5 KB)

Supplementary Material 2 (DOCX 918 KB)

## Data Availability

All data supporting the findings of this study are available within the paper and its Supplementary Information.
