# Supplementary material for: High resolution mapping and development of diagnostic KASP markers for FHB resistance QTL on chromosome 1B in an elite soft red winter wheat cultivar ‘Jamestown’
Source: Mol Breed. 2026 May 28;46(6):57. doi: 10.1007/s11032-026-01680-7 (PMC13219567; doi:10.1007/s11032-026-01680-7)
Supplement: Supplementary file 1 — Supplementary Material 1 (XLXS 16.5 KB) [file 11032_2026_1680_MOESM1_ESM.docx]

**SUPPLEMENTARY MATERIALS**


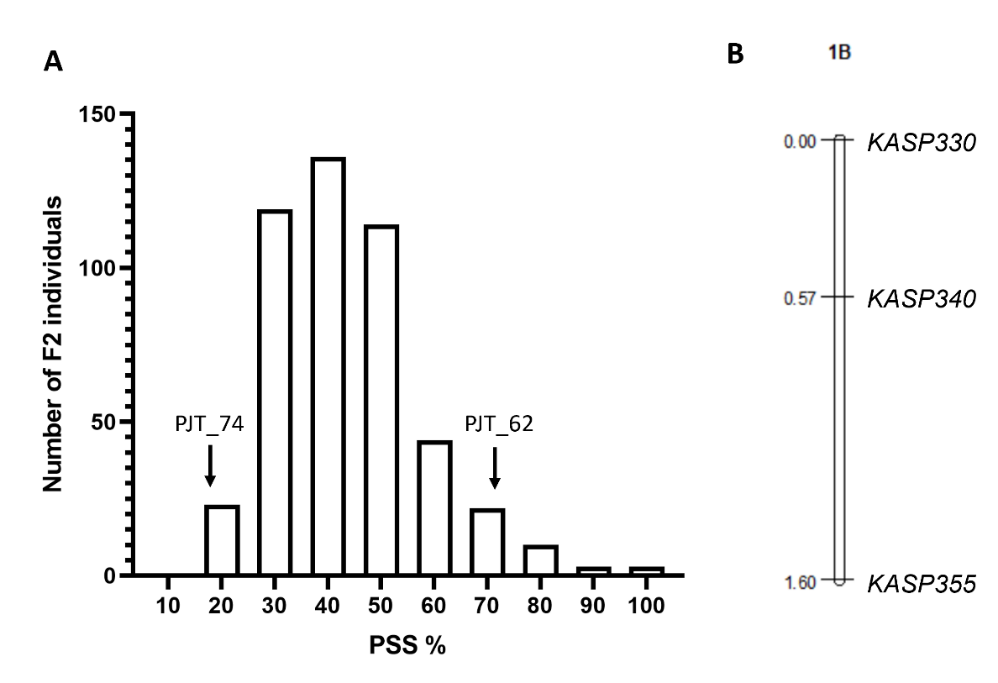


**Supplementary Figure 1. Phenotype and genotype analysis of the PJT_62/PJT_74 F_2_ population.** A) Frequency distribution for PSS (%) in the F_2_ population. B) Genetic map derived from the genotyping of F_2_ population using KASP markers developed in the study.


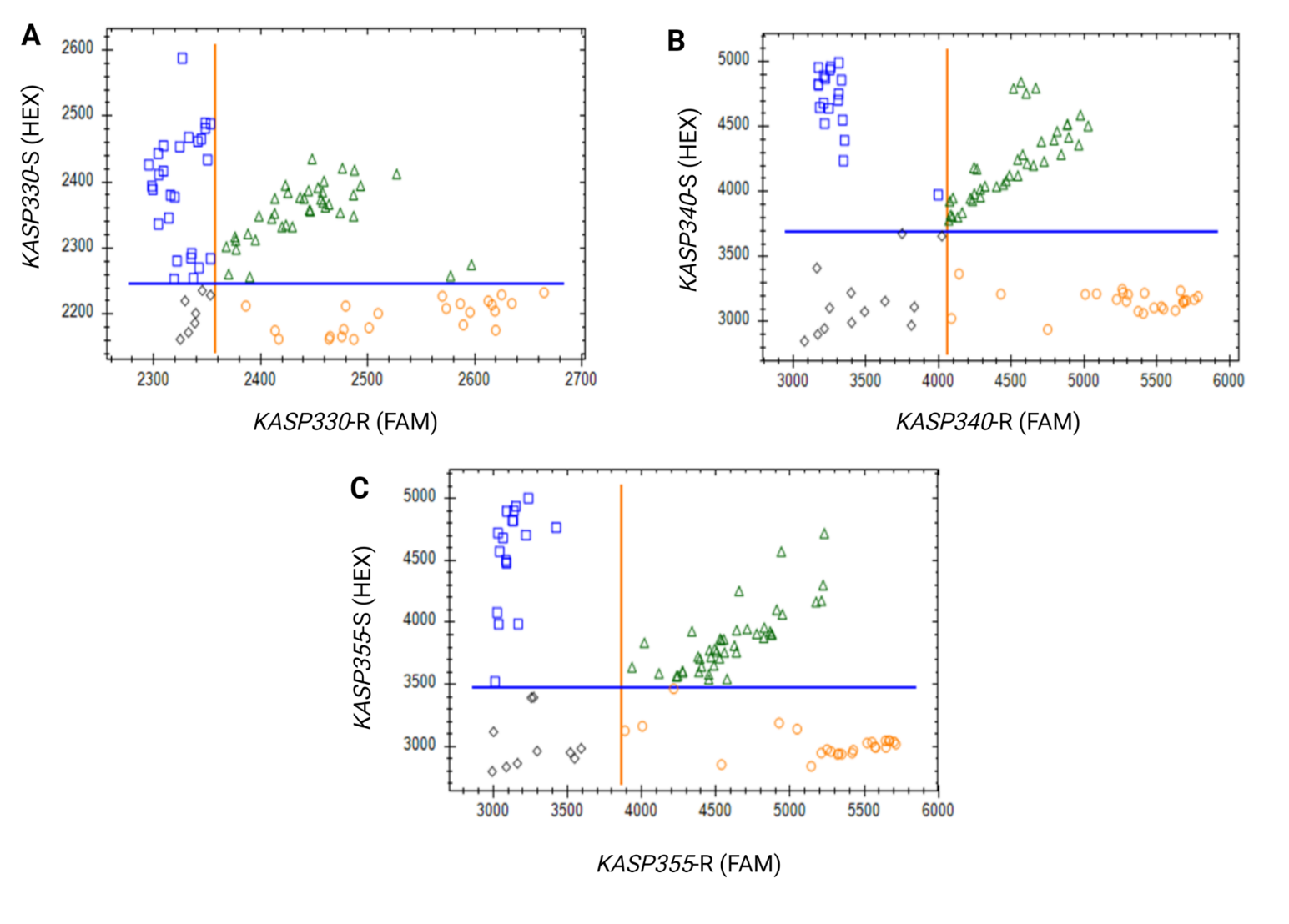


**Supplementary Figure 2.** **KASP assays developed in the study to genotype a high-resolution mapping population.** KASP assay developed for A)- 330 1B_FHB, B) 340_1B_FHB, and C) 355 1B_FHB markers. Orange circles: homozygous for JT allele; Blue squares: homozygous for PR allele; Green triangle: heterozygous with JT/PR alleles; Black diamonds: no template controls (NTC) and nulli 1B DNA.


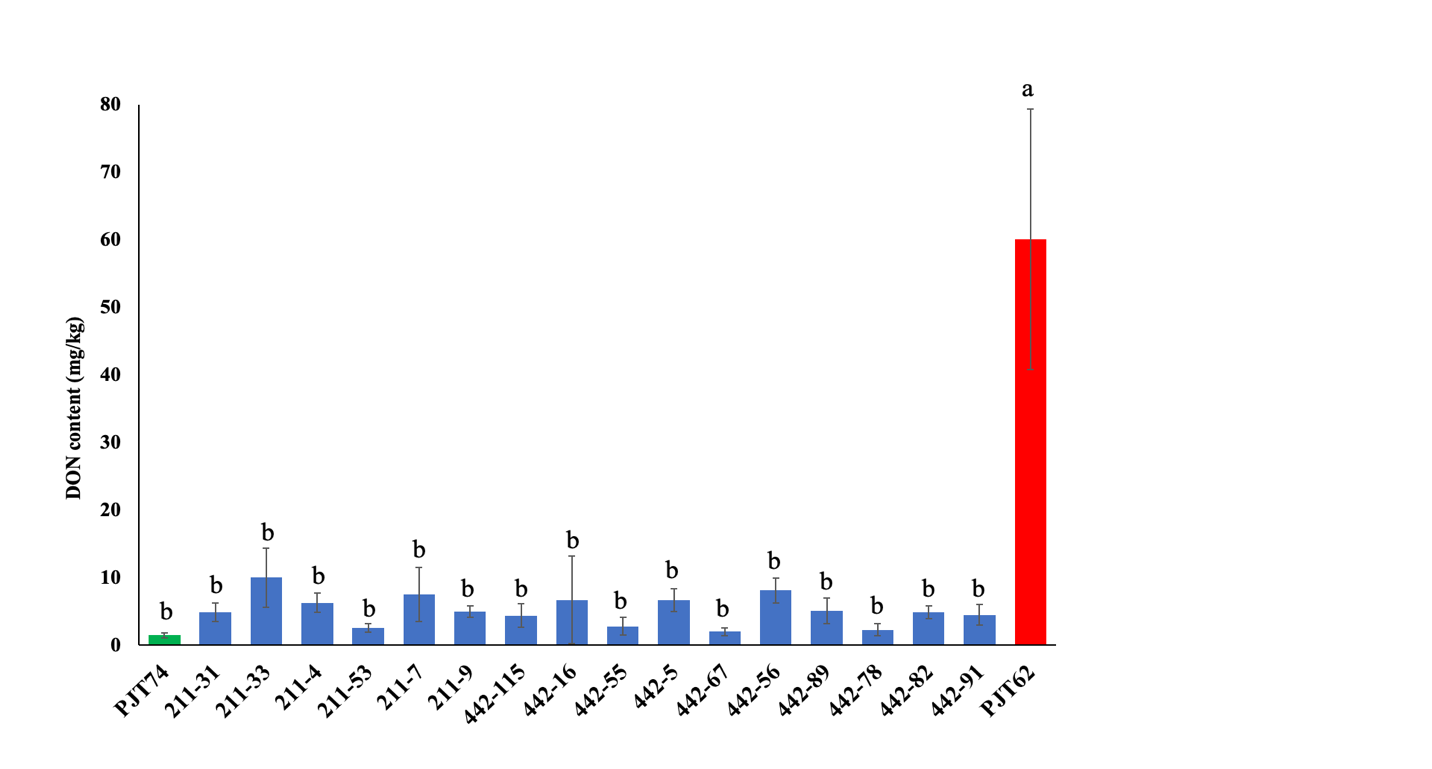


**Supplementary Figure 3. Phenotyping of recombinant F_2_ individuals and their homozygous recombinant F_3:4_ progenies.** DON content of F_3:4_ plants (12 replicates each) and the parents (PJT_62 and PJT_74). Bars with same letter in each panel are not statistically significantly different from each other at p value <0.05.


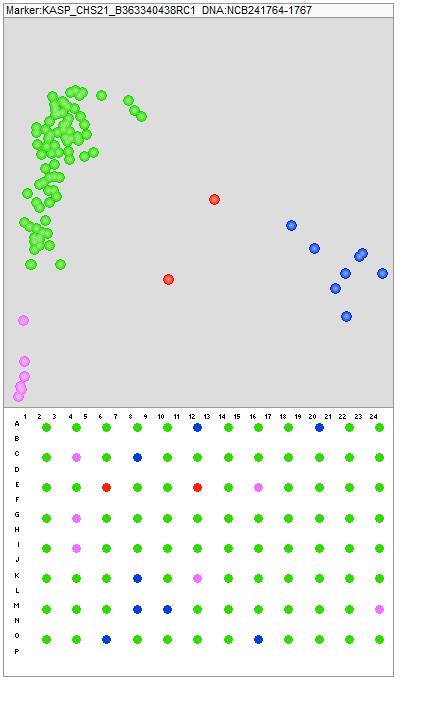

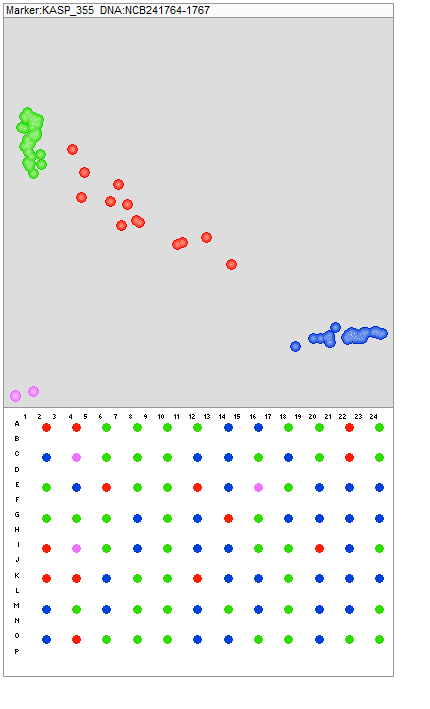

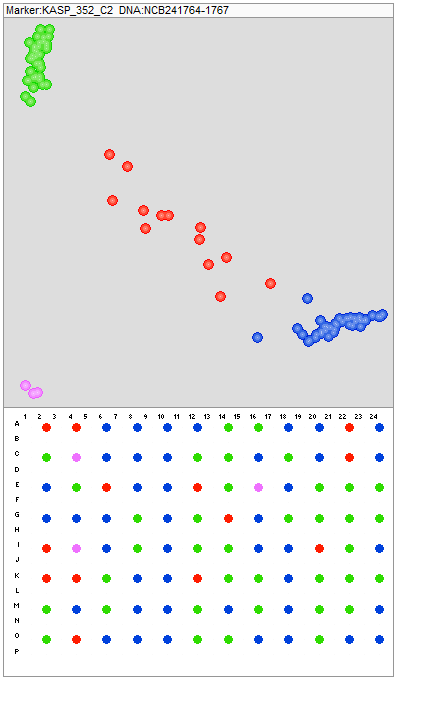


KASP352-R (HEX)

KASP352-S (FAM)

KASP355-R (FAM)

KASP355-S (HEX)

KASP357-R (HEX)

KASP357-S (FAM)

**A**

**B**

**C**

**Supplementary Figure 4.** **KASP validated on 2025 Uniform Nurseries.** KASP assay developed for A) 352 1B_FHB, B) 355_1B_FHB, and C) 357 1B_FHB markers. Green and blue circles represent homozygous allele calls: red circles represent heterozygous/heterogeneous allele calls. JT allele green for 352 and 357, JT allele blue for 355. Pink circles represent non-template controls or missing data.

**Supplementary Table 1.** Sequences of genome-specific polymorphic markers developed in this study

| Marker name | Forward sequence 5'-3' | Reverse sequence 5'-3' | Physical coordinates on 1B | Gene ID |
| --- | --- | --- | --- | --- |
| 330_1B_FHB | CAGGGTGTAAATGCACTGTGT | GAAGAAATTATCCGTGCTTGTTG | 330503665- 330509502 | *TraesCS1B02G183800* |
| 336_1B_FHB | TCTGTCAAACCGCAATGAAG | AGATGAAAAGCCTGGGGG | 336202986- 336205768 | *TraesCS1B02G187900* |
| 340_1B_FHB | CCTGGATCTAGTCATGAAACTTG | CACCAAATGCTAATGGCTCA | 340463564- 340465967 | *TraesCS1B02G189800* |
| 349_1B_FHB | TGCTCTGATCCACCAAATACCAA | GCGACAGAGATGGCCTGCA | 349569330- 349570806 | *TraesCS1B02G194500* |
| 352_1B_FHB | TAGTCAATTGTCCACGCCACAT | GAGTTGAGGATGCCTATCCTTCT | 352389744- 352393541 | *TraesCS1B02G196000* |
| 355_1B_FHB | TTTCCATGTAACCTCGCAAAGA | ACCAGAACAAAACCACTGAGCAC | 355155044- 355157167 | *TraesCS1B02G197600* |
| 357_1B_FHB | AGGTGCCACTTCAAACCTCG | CGAAAGTTCATTGAGCCGA | 357205847- 357209219 | *TraesCS1B02G199000* |
| 364_1B_FHB | TTAAGCTGGCCAAATACTGTTTA | CTCATGGCCTATGTACGTCCG | 364289869- 364294526 | *TraesCS1B02G202700* |
| 375_1B_FHB | ATAGCTACTTTTGCGATTCCAGG | GACCTGGGTAACCATTCCA | 375812152- 375817099 | *TraesCS1B02G207800* |
| 380_1B_FHB | CGCTGCCACTCCTCCTTC | AACAAATTCATCCGTTTAGCTCC | 380435896- 380440076 | *TraesCS1B02G209500* |
| 395_1B_FHB | AGTCAAAATCTGGATCGCCTCTA | GCTTCGACCATGATCACATT | 395258203- 395260226 | *TraesCS1B02G218100* |
| 450_1B_FHB | CGCTGCGCTCTGTCACAT | GATTGACAGCACATACATGAGG | 450608891- 450612736 | *TraesCS1B02G255800* |
| 491_1B_FHB | TGCTTCACCGATTCTCCGA | GAACATGCAAGAAAAGATACCAC | 491081622- 491087500 | *TraesCS1B02G282200* |
